# Apoptosis Signal Regulating Kinase 1 (ASK1): Potential as a Therapeutic Target for Alzheimer’s Disease

**DOI:** 10.3390/ijms15022119

**Published:** 2014-01-29

**Authors:** Juhyun Song, Kyung Ah Park, Won Taek Lee, Jong Eun Lee

**Affiliations:** 1 Department of Anatomy, Yonsei University College of Medicine, Seoul 120-752, Korea; E-Mails: sjh1008@yuhs.ac (J.S.); KAPARK@yuhs.ac (K.A.P.); INSKULL@yuhs.ac (W.T.L.); 2 BK21 Plus Project for Medical Sciences, Yonsei University College of Medicine, Seoul 120-752, Korea

**Keywords:** apoptosis signal regulating kinase 1 (ASK1), Alzheimer’s disease (AD), oxidative stress, endoplasmic reticulum (ER) stress, Aβ neurotoxicity, tau protein phosphorylation, insulin signal transduction

## Abstract

Alzheimer’s disease (AD) is the most common form of dementia, characterized by a decline in memory and cognitive function. Clinical manifestations of AD are closely associated with the formation of senile plaques and neurofibrillary tangles, neuronal loss and cognitive decline. Apoptosis signal regulating kinase 1 (ASK1) is a mediator of the MAPK pathway, which regulates various cellular responses such as apoptosis, cell survival, and differentiation. Accumulating evidence indicates that ASK1 plays a key role in the pathogenesis of neurodegenerative disorders such as Huntington’s disease and AD. Of particular interest, ASK1 is associated with many signaling pathways, which include endoplasmic reticulum (ER) stress-mediated apoptosis, Aβ-induced neurotoxicity, tau protein phosphorylation, and insulin signal transduction. Here, we review experimental evidence that links ASK1 signaling and AD pathogenesis and propose that ASK1 might be a new point of therapeutic intervention to prevent or treat AD.

## 1. Introduction

Alzheimer’s disease (AD) is a neurodegenerative disease characterized by neuronal loss, aggregation of senile plaques derived from amyloid beta (Aβ) peptides, abnormal phosphorylation of tau protein and cognitive decline in the hippocampus or cortex [1,2]. Reactive oxygen species (ROS) production and activation of c-Jun *N*-terminal kinases (JNKs) are involved in many pathological mechanisms in AD [3]. Apoptosis signal-regulating kinase 1 (ASK1) is a protein kinase of the mitogen-activated protein kinase kinase kinase (MAPKKK) family that activates the JNK and p38 MAPK signaling cascades [4,5]. ASK1 is related to various cellular responses including apoptosis, cell survival, and differentiation [6,7]. ASK1 is activated in response to various stresses including tumor necrosis factor (TNF), endoplasmic reticulum (ER) stress, and H_2_O_2_ [4,5,8–10]. In addition, Aβ leading to AD pathology [11] can activate ASK1 that is required for ROS and ER stress-induced JNK activation [12–15]. Insulin like growth factor-1 receptor (IGF-IR) signaling suppresses the ASK1 mediated activation of JNK/p38 pathway. Insulin-like growth factor-1 (IGF-I) can suppress apoptosis, interfere downstream of tumor necrosis factor receptor (TNF-R) activation [16] and block the ASK1 mediated JNK activation by Aβ [17]. The activation of ASK1 also leads to tau phosphorylation that aggravates AD pathology [18]. Therefore, the deterioration of central nervous system (CNS) insulin receptor functions is related to the pathogenesis of sporadic Alzheimer’s disease [19–23]. The cognitive decline is involved in brain insulin dysfunction [24]. ASK1 is involved in insulin signal transduction through TNF-α-induced JNK signaling [25]. In conclusion, ASK1 is associated with various mechanisms, which include cell death, Aβ neurotoxicity, abnormal phosphorylation of tau protein and impaired insulin signal transduction. Hence, ASK1 is involved in mechanisms related to AD pathology.

## 2. ASK1 and Oxidative Stress

**Figure 1.**
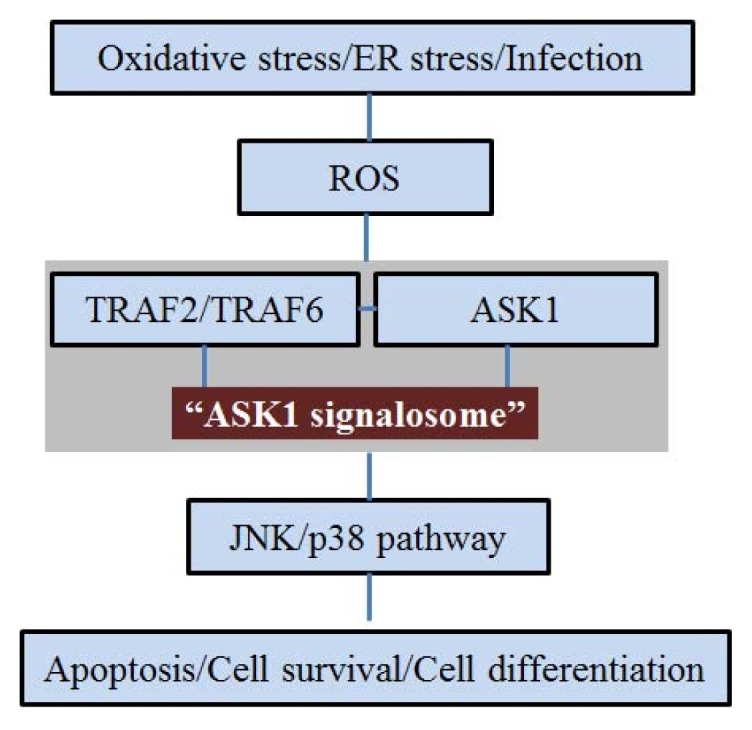
Schematic representation of the relationship between apoptosis signal regulating kinase 1 (ASK1) and c-Jun *N*-terminal kinases (JNK)/p38 pathway activated by various stresses. Various stresses including oxidative stress, ER stress, and bacterial infection, generate reactive oxygen species (ROS). ASK1 activates by forming ASK1 signalosome with TNF receptor-associated factor (TRAF)2/TRAF6. The ASK1 signalosome induces the JNK/p38 pathway and regulates a variety of cellular signal pathways including cell death, cell survival, and the cell differentiation pathway.

The various pathologies in AD are associated with neuronal cell death by oxidative stress. ROS are produced as part of normal cellular metabolic activity. However, excessive production of ROS under oxidative stress causes cell death via apoptosis. MAP kinase signaling involves pathways linking ROS. ASK1 is a MAPKKK and activates both the mitogen-activated protein kinase kinase 4 (MKK4)/MKK7-JNK pathway and MKK3/MKK6-p38 pathway [26]. ASK1 is activated in response to various stresses including TNF, ER stress, and H_2_O_2_ [4,5,8–10,27]. Tobiume *et al*. demonstrated that the activation of the JNK/p38 pathway is attenuated in fibroblasts from ASK1-knockout mice after H_2_O_2_ treatment [5]. The ASK1 activation is regulated by multiple steps including dimerization, phosphorylation, and protein-protein interactions [8,28–31]. Thioredoxin (TRX), which regulates the cellular reduction and oxidation (redox) status, is bound directly to the *N*-terminal region of ASK1 [15]. In the oxidative stress state, ROS induce dissociation of Trx from ASK1. ASK1 is subsequently activated by inducing the oligomerization and the phosphorylation of a critical threonine residue [15,32]. A recent study using gel filtration column chromatography demonstrated that ASK1 constitutively forms the ASK1 signalosome as a high molecular mass complex [33]. The ASK1 signalosome forms a molecular mass complex by recruiting at least two TNF receptor-associated factor (TRAF) family proteins, TRAF2 and TRAF6, which appear to stabilize the complex and promote the activation of ASK1 phosphorylation [33]. Also, in the oxidative stress state, the attenuation of TNF-α expression in the cells isolated from ASK1-knockout mice suggests that ASK1 may act as a regulator of cytokine [5]. ASK1 is associated with TNF-α-induced apoptosis cascades [4,5,26]. To sum up, ASK1 is activated by forming “ASK1 signalosome” with TRAF family proteins in response to oxidative stress and ASK1 is involved in the TNF-α-induced apoptosis pathway (Figure 1).

## 3. ASK1 and ER Stress

The ER stress is caused by the accumulation of unfolded and misfolded proteins in the ER lumen and triggers multiple signals leading to translational and transcriptional apoptosis [34,35]. The ER stress is related to neuronal death occurring in AD [14,36–38]. Mutations of presenilin-1 (PS1) located in the ER are the most common finding in patients with AD. Cells that express PS1 mutants have been reported to be more sensitive to ER stress compared to normal cells [36,38–40]. Based on this relationship, ER stress is an important point to study AD pathology. ASK1 is activated in response to ER stress [4,41] and is required for ER stress-mediated apoptosis [15]. Inositol-requiring enzyme 1 (IRE1) is associated with neuronal death related to ER stress [42] and specifically combines with ASK1. ER stress induces formation of an IRE1-TRAF2-ASK1 complex and activates the ASK1-JNK pathway [15]. The TRAF2-ASK1-JNK pathway plays a central role in ER stress-induced apoptosis [15]. Kadowaki *et al*. demonstrated that primary neurons derived from ASK1-knockout mice brain were resistant to ER stress-induced cell death [12]. In conclusion, ASK1 ultimately activates the JNK pathway in ER stress-induced apoptosis as a component of the IRE1-TRAF2-ASK1 cascade.

## 4. ASK1 and Aβ

**Figure 2.**
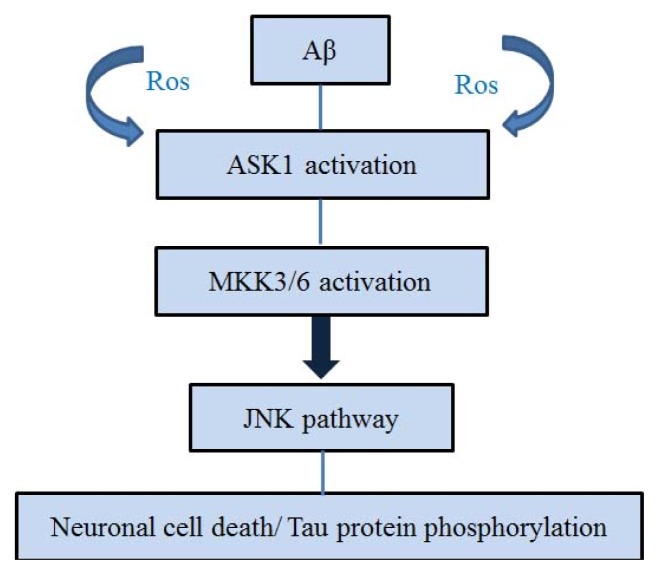
Schematic representation of the relationship between ASK1 and Aβ. Aβ activates ASK1 and MKK3/6 by ROS. Aβ induces the JNK signal pathway, which induces neuronal cell death and phosphorylation of tau protein. Finally, activation of ASK1 by Aβ is associated with neuronal cell death and phosphorylation of tau protein.

The extracellular deposition of senile plaques composed of Aβ and the formation of intracellular neurofibrillary tangles (NFT) caused by abnormal phosphorylation of tau proteins related to regulation of microtubule stability aggravate the progression of AD [43–45]. Aβ is a product of the cleaved amyloid precursor protein (APP) and accumulates as extracellular plaque in the AD brain [46–48]. Kadowaki *et al*. suggested the possibility that Aβ neurotoxicity might be mediated by activation of the ASK1 [12]. Hsu *et al*. demonstrated that the activation of the ASK1-MKK3/6-p38MAPK signaling cascade triggered Aβ-induced cell death in cerebral endothelial cell [49]. Also, tau proteins undergo abnormal phosphorylation and dissociate from microtubules to aggregate into NFTs [50,51]. Hyperphosphorylated tau proteins accumulate to form insoluble paired helical filaments (PHF) within neuronal cell bodies. Aβ induces activation of JNK and phosphorylation of c-Jun [52,53]. Previous studies have demonstrated that Aβ-induced neuronal cell death is inhibited by the expression of a dominant-negative mutant of c-Jun, by the treatment with a JNK inhibitor, or by the disruption of c-Jun or JNK3 [52–54]. Also, Reynolds *et al*. demonstrated that, in the Tg2576/ PS1P264L brain, JNK activation was localized in reactive neurites containing phosphorylated tau proteins. Previous studies have demonstrated that JNK can regulate hyperphosphorylated tau proteins in AD [55]. MKK6 and p38 are recruited by tau, leading to tau phosphorylation at specific and distinct p38-dependent sites [18]. MKK3, MKK4 and MKK6 are JNK-activating MAPK kinases that can be activated by a number of MAPKK kinases including ASK1 [56]. Hashimoto *et al*. demonstrated that the dimerization of the cytoplasmic domains of APP induces ASK1- and JNK-dependent apoptosis in neuronal cells [57,58]. Aβ-induced ASK1 activation is mediated by ROS. Aβ can activate ASK1 that is required for ER-stress-induced JNK activation and apoptosis by ROS [12]. ASK1 is activated by APP dimerization, and both ASK1 and MKK6 are activated by Aβ dimerization of APP [58–60]. Aβ causes an early, strong and transient oxidation of both glutaredoxin-1 (GRX1) and thioredoxin-1 (TRX1). Also, Aβ induces apoptosis by activation of the ASK1 cascade in SH-SY5Y cells [61]. Accordingly, Aβ neurotoxicity is related to the activation of ASK1. Considering the association between Aβ and ASK1, ASK1 might be a potential target for enhancing AD pathology (Figure 2).

## 5. ASK1 and Insulin Signal Transduction

**Figure 3.**
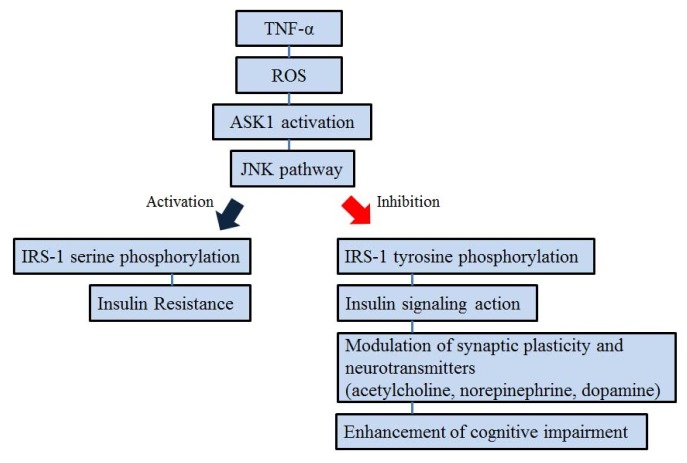
Schematic representation of the relationship between ASK1 and insulin signal transduction. TNF-α triggers the activation of ASK1 mediated JNK signaling. The activation of TNF-α-induced JNK signaling induces serine phosphorylation of insulin receptor substrate (IRS)-1 and insulin resistance whereas the inhibition of TNF-α-induced JNK signaling induces tyrosine phosphorylation of IRS-1 and enhancement of cognitive decline. Finally, ASK1 is related to insulin signal transduction through TNF-α-induced JNK signaling. The inhibition of ASK1 enhances the cognitive decline in AD.

Insulin signaling plays an important role in AD pathology such as cognitive impairment [62,63]. Insulin facilitates glucose uptake in peripheral tissue by binding to the insulin receptor (IR), which belongs to the family of tyrosine kinase receptors. Binding of insulin leads to a rapid auto-phosphorylation on several tyrosine residues that provide docking sites for the insulin receptor substrate (IRS) proteins [64,65]. In the brain, insulin signal transduction is associated with acognitive function, irrespective of changes in peripheral glucose [62,63,66–68]. Several studies have demonstrated that the binding between insulin and IRs regulates the learning and memory functions in brain [62,69–72]. Insulin also modulates the concentration of neurotransmitters associated with cognitive function such as acetylcholine, norepinephrine, and dopamine in the central nervous system (CNS) [73,74]. Additionally, the insulin signaling pathway modulates synaptic plasticity by promoting the recruitment of gamma amino butyric acid (GABA) receptors on post-synaptic membranes, regulating *N*-methyl-d-aspartate receptor (NMDA) receptor conductance and 2-amino-3-(3-hydroxy-5-methylisoxazol-4-yl)propionic acid (AMPA) receptor cycling [75–81]. Tyrosine phosphorylation of the insulin receptor substrates including IRS-1 and IRS-2 is an early and important process of the insulin signal transduction [82,83]. Impaired tyrosine phosphorylation of IRS-1 is correlated with insulin resistance *in vivo* [64,84]. In addition, TNF-α causes insulin resistance through attenuation of IR signaling [64]. TNF-α triggers the activation of ASK1-mediated JNK signaling. The TNF-α-induced JNK signaling increases in Ser^307^ phosphorylation of IRS-1 and decreases in tyrosine phosphorylation of IRS-1. Finally, the Ser^307^ phosphorylation of IRS-1 through TNF-α induced JNK signaling results in insulin resistance [25,65,84]. In conclusion, the increased tyrosine phosphorylation of IRS-1 enhances insulin signal transduction in modulating of neurotransmitters associated with cognitive function and alleviating cognitive decline in brain. ASK1 is involved in insulin signal transduction through TNF-α-induced JNK signaling. Hence, ASK1 serves as a key factor in modulating insulin signal transduction, and its regulation might enhance cognitive decline in AD (Figure 3).

## 6. Conclusions

AD is characterized by neuronal loss, Aβ accumulation, abnormal phosphorylation of tau protein, and cognitive decline in the hippocampus or cortex. ASK1 activates by forming an “ASK1 signalosome” with TRAF family proteins and activates the JNK signaling pathway in response to oxidative stress and ER stress. In addition, Aβ neurotoxicity is associated with the activation of ASK1, and ASK1 is involved in the phosphorylation of tau protein via JNK signaling. Moreover, ASK1 is associated with insulin signal transduction, an important signaling component in cognitive decline. The inhibition of ASK1 induces tyrosine phosphorylation of IRS-1 and prevents the cognitive decline in the brain. Even though activation of ASK1 has not been reported in the AD brain until now, previous studies have indirectly demonstrated that GRX1 and TRX1 modulating ASK1 [41] decreased in the AD brain [61]. Thus, the apparent association between ASK1 and AD pathology related mechanisms advocates the potential of ASK1 to modify the progression of AD. In conclusion, we suggest that ASK1 in the AD brain be more thoroughly investigated in relation with AD pathology.
